# Delivery of Lipid Nanoparticles with ROS Probes for Improved Visualization of Hepatocellular Carcinoma

**DOI:** 10.3390/biomedicines11071783

**Published:** 2023-06-21

**Authors:** Vera S. Shashkovskaya, Polina I. Vetosheva, Arina G. Shokhina, Ilya O. Aparin, Tatiana A. Prikazchikova, Arsen S. Mikaelyan, Yuri V. Kotelevtsev, Vsevolod V. Belousov, Timofei S. Zatsepin, Tatiana O. Abakumova

**Affiliations:** 1 Vladimir Zelman Center for Neurobiology and Brain Rehabilitation, Skolkovo Institute of Science and Technology, 121205 Moscow, Russia; 2 Institute of Translational Medicine, Pirogov Russian National Research Medical University, 117997 Moscow, Russia; 3 Center for Molecular and Cellular Biology, Skolkovo Institute of Science and Technology, 121205 Moscow, Russia; 4 Federal Center of Brain Research and Neurotechnologies, Federal Medical Biological Agency, 119435 Moscow, Russia; 5 Shemyakin-Ovchinnikov Institute of Bioorganic Chemistry, 117997 Moscow, Russia; 6 Department of Chemistry, M. V. Lomonosov Moscow State University, 119991 Moscow, Russia; 7 Koltsov Institute of Developmental Biology of Russian Academy of Sciences, 152742 Moscow, Russia

**Keywords:** reactive oxygen species, hepatocellular carcinoma, HyPer7, hydrocyanine, lipid nanoparticles

## Abstract

Reactive oxygen species (ROS) are highly reactive products of the cell metabolism derived from oxygen molecules, and their abundant level is observed in many diseases, particularly tumors, such as hepatocellular carcinoma (HCC). In vivo imaging of ROS is a necessary tool in preclinical research to evaluate the efficacy of drugs with antioxidant activity and for diagnosis and monitoring of diseases. However, most known sensors cannot be used for in vivo experiments due to low stability in the blood and rapid elimination from the body. In this work, we focused on the development of an effective delivery system of fluorescent probes for intravital ROS visualization using the HCC model. We have synthesized various lipid nanoparticles (LNPs) loaded with ROS-inducible hydrocyanine pro-fluorescent dye or plasmid DNA (pDNA) with genetically encoded protein sensors of hydrogen peroxide (HyPer7). LNP with an average diameter of 110 ± 12 nm, characterized by increased stability and pDNA loading efficiency (64 ± 7%), demonstrated preferable accumulation in the liver compared to 170 nm LNPs. We evaluated cytotoxicity and demonstrated the efficacy of hydrocyanine-5 and HyPer7 formulated in LNP for ROS visualization in mouse hepatocytes (AML12 cells) and in the mouse xenograft model of HCC. Our results demonstrate that obtained LNP could be a valuable tool in preclinical research for visualization ROS in liver diseases.

## 1. Introduction

Reactive oxygen species (ROS) are chemically reactive molecules with various essential functions in living organisms and include hydroxyl radical (∙OH), superoxide anion (O_2_∙^−^), singlet oxygen (^1^O_2_), and hydrogen peroxide (H_2_O_2_) [1,2]. Under normal physiological conditions, the production of ROS is essential for the cells to defend against pathogens and to promote growth and death. However, the increased production of ROS leads to the damage of many biomolecules, apoptosis, and cell arrest [3,4,5]. Most cellular H_2_O_2_ is produced spontaneously or catalytically by superoxide dismutase from (O_2_∙^−^) [6]. H_2_O_2_ is relatively stable in the aqueous solution. It is a potent redox signaling molecule and also can facilitate transmembrane transport [7].

Various methods have been developed to visualize ROS, based on spectrophotometry, fluorescence, chemiluminescence, and electron spin resonance [8,9]. Fluorescence imaging methods are widely used and allow continuous monitoring of the ROS-associated molecules in situ in living cells on a real-time scale using fluorescence-based probes [9]. The most common probes in biomedical research are non-selective sensors based on a leuco-form of fluorescent dyes, which can light up when oxidized by ROS to parental fluorescein, rhodamine, or hydroethidine. However, there are numerous limitations for in vivo applications of these probes critically reviewed in publications [10,11,12,13]. Alternative fluorochromes with emission maxima in the near-infrared region offer low phototoxicity to the cells, low autofluorescence, and good tissue penetration, making them attractive for imaging in tissues [11]. Among these, cyanine dyes are particularly interesting due to their physical and chemical properties and their ability to react with intracellular and extracellular ROS. Genetically encoded sensors are another class of ROS-sensitive probes, e.g., HyPer7. It is a selective ratiometric sensor that allows monitoring H_2_O_2_ concentration, and it can be easily visualized by fluorescence microscopy. Although these probes were used to detect ROS in vivo in several studies, it is still challenging to deliver them to specific organs or tissues [14,15,16]. To prevent early oxidation and increase blood circulation time, cyanine dyes and genetically encoded sensors, such as HyPer7, can be loaded or conjugated to nanoparticles for better visualization of ROS in vivo. Kim et al. showed that hydrocyanine conjugated to chitosan-functionalized pluronic-based nanocarriers can detect ROS in tumor sites by fluorescence and show fluorescence up to two days after injection [14]. Another study showed that the nanoencapsulation of sensors could improve detection or imaging of H_2_O_2_ in biological systems [17]. Hammond et al. developed a novel nanoprobe based on dye encapsulation with improved sensor function for intracellular measurement of H_2_O_2_ [18]. Lipid nanoparticles (LNPs), possessing excellent biocompatibility and low toxicity, are a clinically approved small interfering RNA (siRNA) delivery system for liver diseases, so we decided to use this technology to deliver fluorescent probes for detection of ROS in hepatocellular carcinoma (HCC).

HCC is one of the most malignant and common diseases and is the third leading cause of cancer death [19]. The common mechanism of hepatocarcinogenesis is chronic inflammation associated with severe oxidative stress [20]. It is evident that ROS play a pathogenetic role in the progression of HCC, as they can stimulate the growth of cancer cells [21]. To assess the HCC, noninvasive imaging tools such as magnetic resonance imaging (MRI), ultrasound (US), and computed tomography (CT) are currently used. US is a well-suited tool for diagnosing HCC due to its cost-effectiveness and accuracy in detecting the focal liver lesions [22]. The introduction of microbubbles contrast agents leads to the improved diagnostic capability of HCC by conventional US [23]. MRI and CT are considered as powerful tools for detection of HCC. Recently, several functioning MRI techniques, such as hepatobiliary contrast agents, have been developed to improve the evaluation of liver lesions [23]. However, early diagnosis of HCC still remains challenging, and more studies are required to develop the imaging tools for detection of HCC. Visualization of ROS in HCC models could help to diagnose the disease at an early stage and investigate new drug delivery systems.

Here, we report on the development of an effective delivery system of HyPer7 and hydrocyanine-5 (hydro-Cy5) formulated in LNPs for ROS visualization in HCC. LNPs have demonstrated successful accumulation in the liver and were able to deliver ROS sensors. We expect that it will become an effective system for the detection of ROS in liver diseases.

## 2. Materials and Methods

### 2.1. Cell Culture

AML12 and Huh-7 cell lines were obtained from ATCC. AML12 cells and the Huh-7 cell line were maintained in a 37 °C humidified incubator with 5% CO_2_ in DMEM/F12 (PanEco, Moscow, Russia) supplemented with 10% fetal bovine serum (FBS) (Capricorn, Ebsdorfergrund, Germany).

### 2.2. Synthesis of Hydro-Cy5-siRNA-LNPs

Hydro-Cy5 dye was synthesized based on starting sulfo-Cy5 dicarboxylic acid (Lumiprobe, Moscow, Russia) based on a previously established procedure [24] and formulated in LNPs as described previously [25].

First, hydro-Cy5 was dissolved in ethanol (10 mg/mL) and mixed with lipids. LNP were obtained by mixing a solution of small interfering RNA to firefly luciferase (siLuc) (0.4 mg/mL) in a microfluidic device in a citrate buffer (10 mM, pH 3.0) and an ethanol solution of a mixture of hydrocyanine (3:1 by volume) and lipids and lipidoids (C12-200, 1,2-dystearoyl-CH-glycero-3-phosphocholine, 1,2-dimyristoyl-CH-glycero-3-phosphoethanolamine-N-[methoxy(polyethylene glycol)-2000] and cholesterol) as described previously [25]. The molar ratio of hydro-Cy5:siLuc was 1:1 (hydro-Cy5-LNP_1) or 2:1 (hydro-Cy5-LNP_2).

For biodistribution studies, we used cyanine 5.5 dye (instead of leuco-form of hydrocyanine 5) to obtain fluorescently labeled LNP with different diameters (110 nm and 170 nm); the molar ratio for cyanine 5.5:Luc was 1:10.

### 2.3. Synthesis of LNPs with Plasmid Encoding HyPer7

For HyPer7 construct generation, EGFP was removed from pAAV.TBG.PI.eGFP.WPRE.bGH (addgene #105535, Watertown, MA, USA) using NotI and HindIII restriction enzyme sites. Then, amplified from pCS2+HyPer7-NES (addgene #136467, forward primer: 5′-ATATATGCGGCCGCGCCACCATGCACCTGGC-3′, reverse primer: 5′-ATATATAAGCTTTTACAGGGTCAGCCGCTCCA-3′), a cytosolic version of HyPer7 with an added nuclear exclusion sequence (NES) was cloned into the prepared backbone.

Two methods were used to obtain lipid nanoparticles with plasmid DNA (pDNA): manual formulation and microfluidics. In manual formulation, LNPs were obtained by mixing solutions of pDNA (0.1 mg/mL) in citrate buffer (10 mM, pH 3) and lipids in ethanol (3:1 by volume) by rapid addition of ethanol in the buffer and further suspension. The lipids (C12-200, dioleoylphosphatidylethanolamine—DOPE or dioleoylphosphatidylcholine—DOPC, cholesterol, PEG-lipid) were mixed in ethanol in a molar ratio of 35:16:46.5:2.5. The weight ratio of DNA/C12-200 is 1:10. The mixture was incubated for 10 min at room temperature to complete particle self-assembly. The nanoparticle suspension was diluted in a PBS, dialyzed overnight in 2000 molecular weight dialysis cassettes against 500 times the volume of PBS at pH 7.4, and filtered through a polyethersulfone syringe filter (0.22 μm) (Table S1).

Another formulation method of LNPs was focused on mixing solutions of pDNA and lipids (3:1 by volume) using the NanoAssemblr^®^ Benchtop microfluidic device (Precision NanoSystems, Vancouver, BC, Canada). The molar ratio of lipids in the mixture, the mass ratio of DNA/C12-200, and the concentration of pDNA were used the same as in the manual formulation. The solution mixing range was 10 mL/min. The use of microfluidic formulation allows the mixing rate to be adjusted by the flow rate of each of the solutions. The aqueous and ethanol phases containing dissolved components of nanoparticles are injected into each inlet of the NanoAssemblr cartridge using syringes. Computer control allows the input mixing parameters to be controlled to optimize particle characteristics such as particle size and encapsulation efficiency and eliminate variability.

### 2.4. Characterization of Obtained LNP

Particle sizes were measured and calculated by intensity using Zetasizer Nano ZSP (Malvern Panalytical, Malvern, UK) according to the manufacturer’s protocol [26]. The loading efficiency of pDNA was analyzed by using the Quant-iT™ RiboGreen^®^ reagent (Thermo Fisher Scientific R11491, Waltham, MA, USA) as described earlier [27]. First, a calibration curve was obtained using standard pDNA in the concentration range 0.03–0.2 µg/mL. After that, DNA concentration was measured in obtained nanoparticles before and after disruption with the Triton X-100 buffer. The difference in these values shows the portion of loaded pDNA.

### 2.5. Cytotoxicity of Obtained Lipid Nanoparticles (MTT Assay)

The cytotoxicity of selected LNPs was measured on a culture of mouse hepatocytes (AML12). Cells were seeded in a 96-well plate and grown to 70% confluent monolayer (48 h, 5% CO_2_, 37 °C). The LNPs were diluted in DMEM/F12 (PanEco, Moscow, Russia) with 10% FBS (Capricorn, Ebsdorfergrund, Germany), added to the cells, and incubated for 24 h (5% CO_2_, 37 °C). The next day, the cells were washed with PBS and MTS-reagent was added (Promega, Madison, WI, USA) according to the manufacturer’s protocol. Absorbance at 490 nm was then analyzed. Unexposed cells were used as a control.

### 2.6. Analysis of ROS Activity In Vitro

Analysis of ROS activity in vitro was performed using a fluorescent microplate reader (Varioskan Lux, ThermoFisher, Waltham, MA, USA). We incubated hydro-Cy5-LNPs with 10 µM H_2_O_2_ for 10 min at room temperature and quantified fluorescence at 640/680 emission/excitation filters.

For imaging experiments, AML12 cells were seeded in a 35 mm glass confocal dish. Twenty-four hours after incubation with LNP, cells were washed with 1 mL PBS (PanEco, Moscow, Russia). To determine the response of LNP- hydro-Cy5 to exogenous H_2_O_2_, a solution of H_2_O_2_ was added to a final concentration of 10 µM. Cell imaging was performed using a Nikon Eclipse Ti2 microscope. Fluorescence of LNP- hydro-Cy5 probes was analyzed at 640/680 excitation/emission filters.

### 2.7. Delivery of LNPs to the Liver and Analysis of ROS Activity in Vivo in HCC Model

All experiments were approved and performed in accordance with institutional guidelines of the Koltsov Institute of Developmental Biology of the Russian Academy of Sciences (Approval #60) and Pirogov Russian National Research Medical University (Moscow, Russia, Approval 06/2021).

Biodistribution. For biodistribution experiments we intravenously injected cyanine 5.5-LNP (size 110 nm and 170 nm) and analyzed whole body fluorescence using IVIS Spectrum CT (Perkin Elmer, Waltham, MA, USA) at 5 min, 1, 4, 6, and 24 h after injection at 675/720 excitation/emission filters. Spectral unmixing was performed using Living Imaging 4.5.3 software to separate the tissue autofluorescence signal. For the biodistribution experiment of LNPs loaded with pDNA, we used plasmid-encoding firefly luciferase (Fluc) with an average diameter 110 nm and injected it intravenously (500 µg DNA/kg); after that, we analyzed its luminescence using IVIS Spectrum CT (Perkin Elmer, Waltham, MA, USA) at 3, 6, 9, and 14 days after injection.

HCC model. To induce ROS in the liver, we used an HCC xenograft model. We injected Huh-7-Luc cells (2 × 10^6^ cells in 50% Matrigel^®^) to the nu/nu mice (male, 6–8 weeks old) in the median lobe of liver. Seven days after tumor implantation, we injected the obtained LNPs in PBS, and analyzed its fluorescence using IVIS Spectrum CT. For ex vivo visualization of ROS, animals were anesthetized (Zoletil^®^ (tiletamine + zolazepam) 20 mg/kg, xylazine 0.2 mg/kg) and livers were imaged using 640/680 excitation/emission filters. Photons of tumor and healthy tissue were quantified using Living Image software (Xenogen Corp., Alameda, CA, USA).

### 2.8. Statistical Analysis

GraphPad Prism 8.2.1 (GraphPad Prism Software, Inc., San Diego, CA, USA) was used for statistical analysis. Tukey’s multiple comparison test with 95 % confidence level or two-way ANOVAs were used for statistical analysis. A *p*-value less than 0.05 was considered as significant.

## 3. Results

### 3.1. Synthesis and Characterisation of Lipid Nanoparticles and ROS Analysis In Vitro

Previously, we synthesized two different hydro-Cy5 derivatives and analyzed their sensitivity for ROS detection [7]. In this study, we investigated the possibility of using the free dye after intravenous bolus injection into the blood of healthy animals and animals with HCC, as was previously described in other studies [28,29]. Unfortunately, we observed rapid hydro-Cy5 dye excretion from the body and a strong fluorescence signal in the bladder within 5 min after intravenous injection (Figure S1). Therefore, we decided to encapsulate hydro-Cy5 in LNP to improve targeted delivery to the liver. The zeta potential of hydro-Cy5-LNP is −7.58 mV.

One of the newly developed molecular probes for ROS visualization is the pDNA HyPer7, which encodes the HyPer protein sensitive to H_2_O_2_ and capable of responding to the ROS changes in the cell within seconds [30]. In order to efficiently deliver HyPer7 to the liver in vivo, we also decided to encapsulate it in LNPs, as publications show that LNPs can serve as an efficient delivery system for different molecules [25]. While LNP are clinically approved for siRNA and mRNA delivery, efficient encapsulation and delivery of pDNA requires optimization of composition and molar ratio of lipids. For the synthesis of LNPs with HyPer7, we decided to compare the LNP formulation by using two different helper lipids, DOPE and DOPC. The other lipids used and their proportions were not changed. The manual formulation leads to the formation of particles of uncontrolled size in the diameter range of 120 to 250 nm with a high degree of sample heterogeneity. The main disadvantage of this method is the lack of the possibility to control the parameters of the LNPs using the mixing rate in manual formation. In the case of microfluidic cartridges, the particles have a smaller size, significantly higher efficiency of pDNA loading, and their characteristics are much more reproducible. The polydispersity of LNPs is comparable to that of particles obtained by manual formulation, but the polydispersity index (PDI) values are not critical for using such nanoparticles in vivo (Table 1).

**Figure 1:**
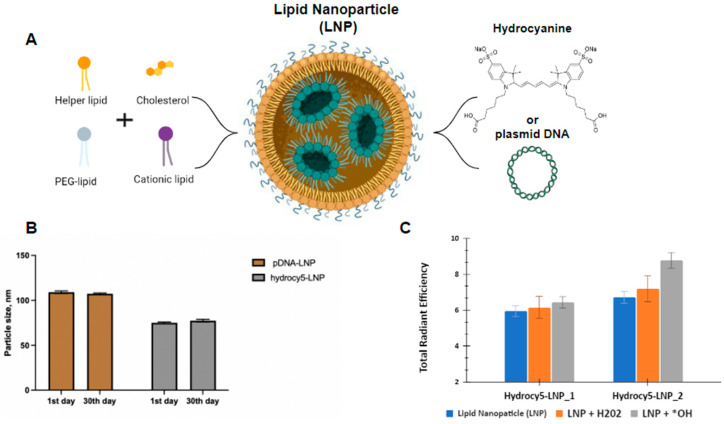
Synthesis and characterization of LNP. (**A**) Schematic representation of the LNPs composition; (**B**) the stability of obtained LNPs; (**C**) fluorescence intensity analysis of LNP with hydrocyanine (LNP_1, LNP_2) before and after interaction with ROS (H_2_O_2_ and hydroxyl radical (*OH)).

Cytotoxicity and stability of the obtained LNPs were then evaluated. The particles were shown to retain their size at 4 °C in the sterile solution (Figure 1B). We evaluated the cytotoxicity of LNP-pDNAs (IC_50_ = 0.429 ng DNA/ml) that is comparable with other studies (Figure S2). We used the similar LNP concentration as in our previous study that demonstrates that the administration of LNP did not result in pathological changes in liver tissue [31]. It is also comparable to the IC_50_ data of previously studied LNPs with siRNAs [32].

We obtained two types of particles: LNP_1, LNP_2 (1× and 2× dye excess to siLuc during synthesis, respectively) with an average diameter of 80 nm. It was shown that the hydro-Cy5 encapsulated in particles didn’t change the fluorescence intensity when interacting with the H_2_O_2_. In contrast, LNP_2 showed a slight fluorescence increase when reacted with highly reactive hydroxyl radical (Figure 1C). These data suggest a high stability of the LNPs and a high degree of dye protection against oxidation after intravenous injection.

Next, we decided to determine optimal amount of dye incoroporated in LNP for the detection using IVIS Spectrum CT. For this purpose, we conjugated LNP with different amount of cyanine 5.5 and showed that the amount of incorporated dye impacts LNP detection in organs (Figure S3) and further efficacy evaluation. So, we proceeded experiments with LNP_2 and tested their efficacy in vitro.

**Figure 2:**
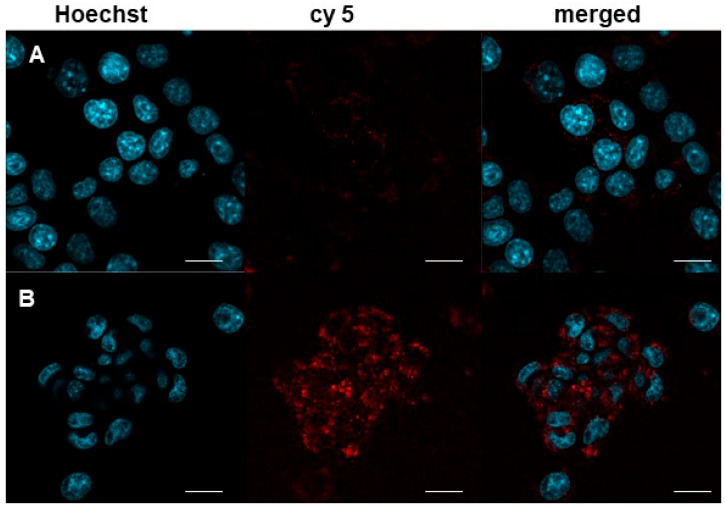
Confocal microscopy images of the AML12 cells incubated with hydro-Cy5-LNP before (**A**) and after (**B**) H_2_O_2_. Nuclei are stained with Hoechst, and hydro-Cy5 is indicated in red color. The scale bar is 50 µm.

Next, we analyzed the efficacy of hydro-Cy5-LNP for ROS visualization on the AML12 cell line in vitro. For this purpose, AML12 were incubated with hydro-Cy5-LNP for 24 h, and then 10 µmol H_2_O_2_ was added to the cells. Confocal images were taken of the AML12 before and after H_2_O_2_ addition. Cell nuclei were stained with Hoechst 33342 (in blue), and the LNPs were imaged in red to monitor oxidation of hydro-Cy5-LNP in the cells. We demonstrated in vitro that hydro-Cy5-LNPs fluorescence increased in the presence of 10 µmol H_2_O_2_, which is relevant to ROS changes in vivo during liver inflammation (Figure 2).

### 3.2. Intravital Delivery of ROS Sensor-Lipid Nanoparticles and Visualization of Hepatocellular Carcinoma

**Figure 3:**
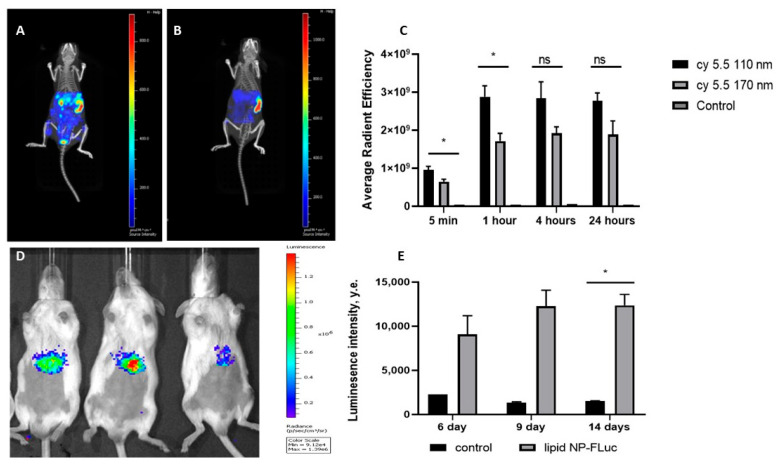
Biodistribution of 110 nm (**A**) and 170 nm (**B**) cy5.5-labeled LNPs in 24 h after injection and the quantification of fluorescence intensity in the liver in 5 min, 1, 4, and 24 h after injection (**C**); the luminescence intensity (**D**) in mice in 9 days after intravenous injection of LNPs loaded with pDNA Fluc and quantification of luminescence intensity at 6th, 9th, and 14th days after injection of LNPs (**E**). Control indicates a healthy mouse. Data are presented as mean ± SD, *n* = 4 (ns—not significant, *—*p* < 0.05).

To preliminarily evaluate the size effect on particle delivery efficiency, we intravenously injected cyanine 5.5-labeled LNPs to the FvB/N mice. Whole-body fluorescence images were analyzed using the IVIS Imaging CT system in 15 min, 1, 4, and 24 h of injection of the 110 and 170 nm particles (Figure 3C). It was shown that LNPs with an average diameter of 110 nm demonstrated preferable accumulation in the liver after 24 h (Figure 3A), while LNP with size of 170 nm mainly accumulated in the spleen (Figure 3B). So, we decided to use 110 nm LNP for further experiments.

Next, we evaluated the efficiency of pDNA-LNP delivery to mouse liver cells. LNPs containing pDNA to Fluc with a diameter 110 nm were injected intravenously and the luminescence intensity was analyzed 3, 6, 9, and 14 days after injection. It was shown that the particles effectively transfected the liver after 6 days up to 14 days without reinjection (Figure 3D,E). These results confirm the possibility of effective pDNA delivery using LNPs.

**Figure 4:**
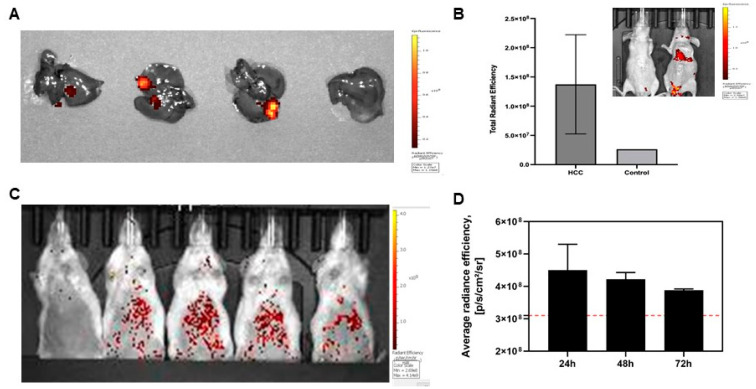
Images of liver tissues of the mice with HCC after administration of LNPs loaded with hydrocyanine: ex vivo imaging using IVIS Spectrum CT 3 h after injection (**A**) and quantification of fluorescence intensities (**B**); (**C**). Images of mice after injection of LNPs loaded with HyPer7; (**D**). Quantification of fluorescence intensities after injection of LNPs loaded with HyPer7; red line indicates control. Data are presented as mean ± SD, *n* = 3.

To evaluate the efficacy of ROS imaging using the developed LNPs, we decided to use the HCC mouse model. We demonstrated the efficacy of ROS imaging in a mouse model of HCC after the administration of hydrocyanine nanoparticles. It was shown that these particles effectively accumulate in liver tissue and specifically fluoresce in the area of the pathology (Figure 4).

We observed an insignificant increase in fluorescence intensity (1.5 times in comparison with control) in 48 h after LNP-HyPer7 injection (Figure 4C). Similar results were observed 10 days after injection of HyPer7-LNP (Figure S4). However, the injection of obtained LNPs into the HCC-bearing mouse did not significantly change the fluorescent signal. These results demonstrate persistent limitations of HyPer7 for in vivo imaging that requires either different pDNA delivery methods (viral or hydrodynamic) or other detection methods.

## 4. Discussion

ROS play an important role in many biological pathways, but long-term inflammation leads to the overproduction of ROS, causing oxidative stress and the development of many pathologies, including liver diseases [11,33]. Cancer cells have elevated levels of ROS that promote tumor growth and are responsible for the development of numerous liver diseases, including alcoholic liver diseases, nonalcoholic fatty liver diseases, hepatic fibrosis, and hepatitis C virus [5]. ROS activate hepatic stellate cells, increasing the inflammation and initiating liver fibrosis [34]. One of the most common malignant liver diseases is HCC, which is associated with many cellular processes and affects the metabolism of liver cells, resulting in increased ROS production. For diagnosis and monitoring of different liver diseases and namely HCC, visualization of ROS remains an important tool for preclinical study. Here, we demonstrate the development of two types of LNPs loaded with ROS sensors—pDNA HyPer7 and the hydro-Cy5 dye, which can be used for the detection and monitoring of ROS in HCC.

LNP technology has played the central role in the development of the first three approved drugs, namely Patisiran as siRNA medicine and Tozinameran and Elasomeranas as mRNA vaccines [35]. Patisiran (ONPATTRO^®^) is used for the treatment of polyneuropathies associated with the hereditary disease transthyretin-mediated amyloidosis [36]. The two recent FDA approval of COVID-19 based modified mRNA vaccines are formulated in LNPs [37]. The LNPs formulation is a promising strategy for DNA/RNA delivery (pDNA, mRNA, siRNA) to the different tissues, and it can be effective for the accumulation of ROS-sensitive dyes in the liver and improve the visualization of ROS [38,39]. In our work, we compared the manual formulation of LNPs and formulation by microfluidic device. Manual formulation can be easily conducted while microfluidic technologies allow to produce homogenous-sized LNPs and have accelerated the development of LNP-based nanomedicine [40]. Lopes et al. outlined that microfluidic technologies promote the more stable and uniform LNPs [41]. Unsurprisingly, microfluidic device technology allows production of LNP with high loading efficiency and low PDI, which we observed by pDNA and hydro-Cy5 formulation. We demonstrated that obtained LNPs are stable and retain their size within 30 days, which means that the LNPs do not aggregate over time, which is essential for using them both in vitro and in vivo. Composition of lipids can impact LNP characteristics; however, we did not observe any difference in DNA loading efficiency using two different helper lipids, DOPE and DOPC. This could be explained by the similar chemical structure of both lipids. However, selection between DOPE and DOPC by formulation in LNPs can affect the transfection efficiency both in vitro and in vivo. Zhang et al. found that DOPE-containing LNP formulation enhances the mRNA delivery to the liver [42]. While Kulkarni et al. showed that replacement of DSPC with DOPC increased transfection efficacy with pDNA-LNP in vitro more than 40x times [43]. However, we observed only slight changes in DOPE- and DOPC-LNP-pDNA transfection efficacy in vivo in 24–72 h after intravenous injection. Danaei et al. outlined that the nanoparticles with the PDI value 0.2 and below are most commonly accepted in practice for polymer-based nanoparticle materials [44]. In our work, we used LNPs with PDI values no more than 0.22 obtained by a microfluidic device, which is consistent with the previous studies [45]. Multiple formulation parameters, lipid components and composition, lipid ration, and processing parameters are crucial for LNPs with PDI values less than 0.2 [46].

Genetically encoded probes that are sensitive to the H_2_O are promising approaches for monitoring the production of intracellular H_2_O_2_. The main problem of HyPer and other probes containing the circular permutation is their high sensitivity to the pH. Recently, a new member of the HyPer family, HyPer7, was introduced that is non sensitive to the changes in pH and more sensitive to the H_2_O_2_ [47]. We used HyPer7 in this study for visualization of ROS in vivo that was formulated in LNP for the efficient delivery to the liver. Although, we demonstrated that we can successfully deliver pDNA Fluc to the liver cells, we did not observe the significant fluorescence after HyPer7-LNP injection in comparison with control. Possibly, it might be due to the fact that the one of main disadvantages of green fluorescence probes to be used in vivo models is the low tissue penetration [48]. The low fluorescence intensity of the liver after intravenous injection of HyPer7-loaded LNPs could be also explained by the insufficient delivery method that could be optimized by the other composition of the lipids or amount of pDNA or detection methods. Godbout et al. outlined that it is crucial to modify the composition of the lipids used for formulation in LNPs that will lead to the specific delivery of LNPs to the organs [49]. Additionally, adenovirus-mediated delivery, polymeric nanoparticles, or hydrodynamic transfection could increase the efficacy of HyPer7 delivery. Zamboni et al. demonstrated that polymeric biodegradable poly(beta-amino-ester) nanoparticles enabled high DNA delivery to HCC cells [50]. Additionally, it should be noted that HyPer is generally used for ratiometric imaging of the living cells, so detection method plays an essential role in the efficacy of H_2_O_2_ measurement; in particular, intravital microscopy should be considered [51].

One of the probes that can be used for visualization of ROS are hydrocyanine dyes that are nonfluorescent in leuco form but can be oxidized by hydroxyl radicals and become fluorescent cyanine molecules [52]. Different probes from the hydrocyanine family have different emission wavelengths and can be used for different purposes for in vitro and in vivo studies. Hydrocyanines were used for measuring the ROS production in vivo in different animal models, such as inflammation, but their application is limited due to the low tissue penetration of optical probes [52]. In our work, we used hydro-Cy5 dye that was formulated in LNP to protect it from oxidation in the blood stream and increase accumulation in vivo. To prove the high stability of obtained LNPs, we decided to compare the efficacy of LNPs loaded with two different excess levels of hydro-Cy5. We did not observe significant changes in the fluorescence intensities by adding H_2_O_2_, which proves the high stability of LNPs. We also tested efficacy of hydro-Cy5-LNP in AML12 cells and demonstrated that LNPs increased fluorescence already 5 min after H_2_O_2_ addition compared to non-treated cells, which indicates that hydro-Cy5 encapsulated in LNPs is sensitive to the intracellular H_2_O_2_ and can be used for further in vivo experiments. Therefore, we decided to study the biodistribution of hydro-Cy5-LNPs of two sizes—110 and 170 nm. Although intravenous studies show that the liver is the major organ depot for nanoparticles [42], we observed the accumulation of hydro-Cy5-LNP 170 nm in the spleen but not in the liver compared to the hydro-Cy5-LNP 110 nm, which does not allow us to use it for detection of ROS in HCC. We successfully demonstrated ROS-sensitive detection of tumor tissue in an orthotopic HCC model after intravenous injection of hydro-Cy5-LNP. It should also be noted that this delivery platform could also be used for simultaneous siRNA delivery and ROS visualization.

In conclusion, we synthesized LNPs with ROS-sensitive probes and demonstrated the successful delivery of pDNA-LNP and hydro-Cy5-LNP to the liver. We showed that accumulation of hydro-Cy5-LNP results in ROS-specific visualization of tumor tissue in the mouse model of HCC compared to the control. We believe that our results present a valuable tool to visualize ROS in liver diseases by LNPs loaded with hydro-Cy5 that can be used in preclinical research.

## Figures and Tables

**Table 1:** The characteristics of LNPs obtained by manual and microfluidic formulation.

Formulation	Cationic Lipid	Particle Size, nm	PDI	DNA Concentration in the Particles, ng/µL	DNA Loading Efficiency, %
Manual	DOPE	177.7 ± 48.1	0.18	6.3 ± 1.1	10.8 ± 1.9%
Manual	DOPC	214.3±15	0.2	8	13.6%
Microfluidic	DOPE	120	0.2	34	62%
Microfluidic	DOPC	108 ± 12	0.22 ± 0.02	36 ± 5	64 ± 7%

## Data Availability

The datasets used and analyzed during the study are available from the corresponding author upon request.

## Supplementary Materials

The following supporting information can be downloaded at: https://www.mdpi.com/article/10.3390/biomedicines11071783/s1, Figure S1: The fluorescent images of mice in 5 min (A) and 15 min (B) after administration of unconjugated hydro-Cy5; Figure S2: Cytotoxicity of 110 nm LNPs; Figure S3: Images of organs (organs (heart, lungs, liver, kidneys, spleen) after cy5.5-labeled LNP administration with 1× or 2× dye excess in 4 h and 24 h after injection. Figure S4: Whole body fluorescent images in 10 days after LNP-HyPer7 administration; Table S1: The parameters of LNPs synthesis.
