# Supplementary material for: Delivery of Lipid Nanoparticles with ROS Probes for Improved Visualization of Hepatocellular Carcinoma
Source: Biomedicines. 2023 Jun 21;11(7):1783. doi: 10.3390/biomedicines11071783 (PMC10376883; doi:10.3390/biomedicines11071783)
Supplement: Supplementary file 1 [file biomedicines-11-01783-s001.zip › Table S1.pdf]

Table S1. The parameters of LNPs synthesis.

Category	Parameters
Ionizable lipid	C12-200
Molar lipid ratios (%) C12-200/DOPE/Cholesterol/C14-PEG 2000	35:16:46.5:2.5
Concentration of pDNA in aqueous phase (mg/ml)	0.1
The mass ratio of DNA/C12-200	1:10
Volume of aqueous phase (ml)	3.0
Volume of alcohol phase (ml)	1.0
Total Flow Rate (ml/min)	10.0
Flow Rate Ratio	3:1
