# Supplementary figures and images for: Delivery of Lipid Nanoparticles with ROS Probes for Improved Visualization of Hepatocellular Carcinoma
Source: Biomedicines. 2023 Jun 21;11(7):1783. doi: 10.3390/biomedicines11071783 (PMC10376883; doi:10.3390/biomedicines11071783)

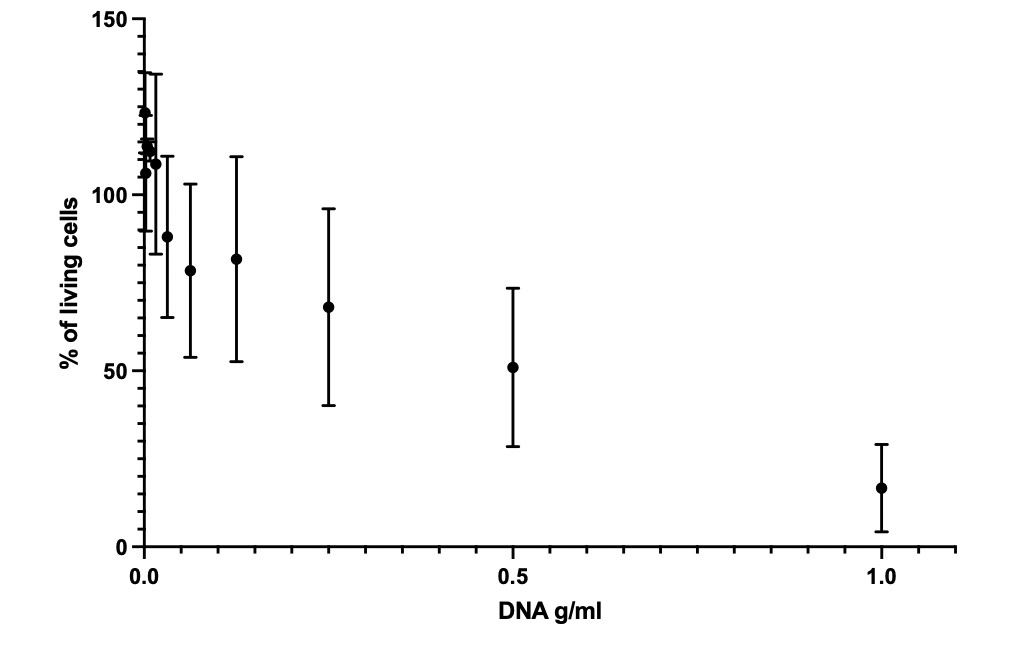

Supplement: Supplementary file 1 [file biomedicines-11-01783-s001.zip › Figure S2. Cytotoxicity of 110 nm LNPs.jpg]

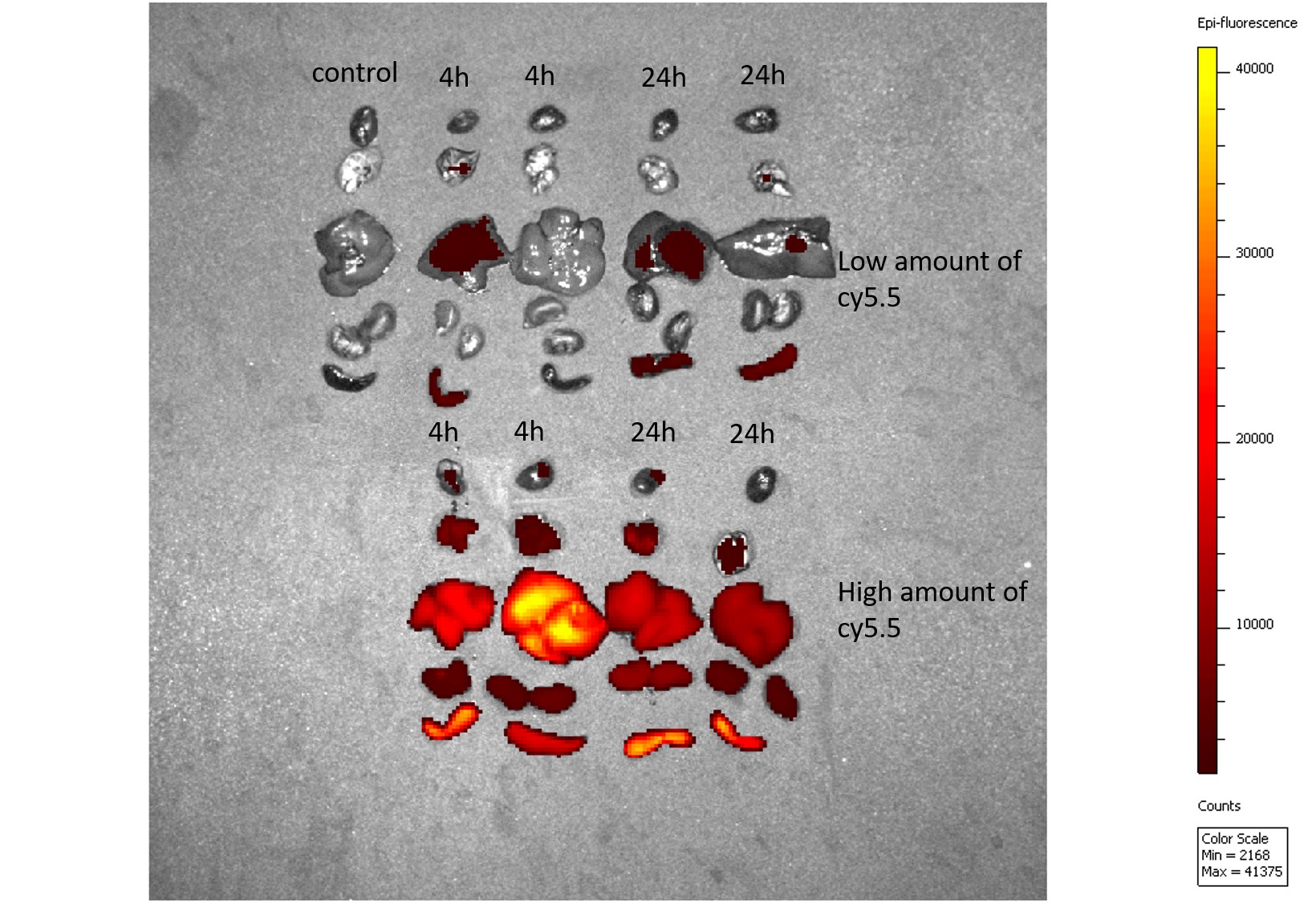

Supplement: Supplementary file 1 [file biomedicines-11-01783-s001.zip › Figure S3. Images of organs (heart, lungs, liver, kidneys, spleen) after cy5.5-labeled LNP administration with 1x or 2x dye exce.jpg]

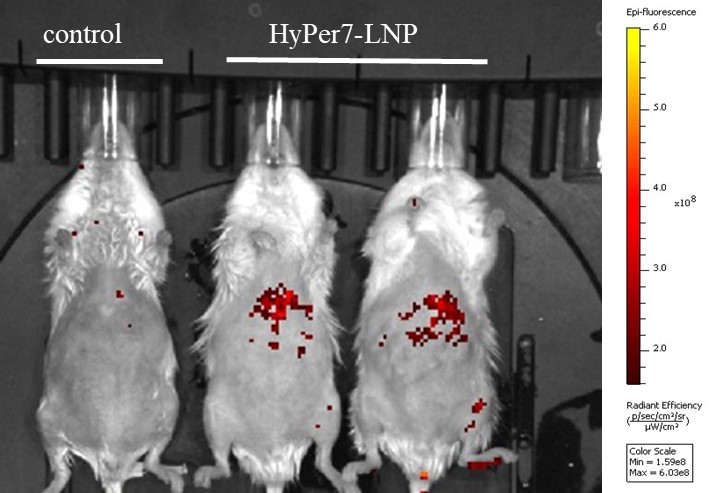

Supplement: Supplementary file 1 [file biomedicines-11-01783-s001.zip › Figure S4. Whole body fluorescent images in 10 days after LNP-HyPer7 administration.jpg]

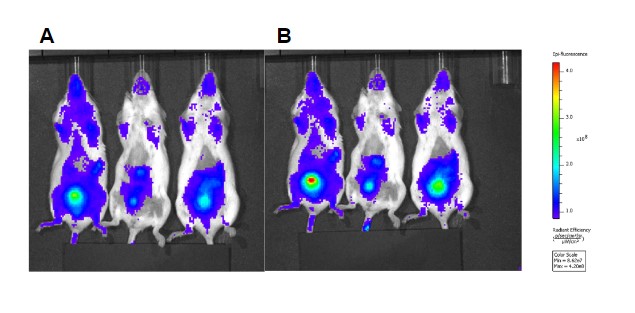

Supplement: Supplementary file 1 [file biomedicines-11-01783-s001.zip › Figure S1. The Fluorescent images of mice in 5 min (A) and 15 min (B) after administration of unconjugated hydrocy5.jpg]
